# Stepping up to the thermogradient plate: a data framework for predicting seed germination under climate change

**DOI:** 10.1093/aob/mcac026

**Published:** 2022-02-25

**Authors:** Justin C Collette, Karen D Sommerville, Mitchell B Lyons, Catherine A Offord, Graeme Errington, Zoe-Joy Newby, Lotte von Richter, Nathan J Emery

**Affiliations:** The Australian PlantBank, Australian Institute of Botanical Science, Australian Botanic Garden, Mount Annan, NSW 2567, Australia; The Australian PlantBank, Australian Institute of Botanical Science, Australian Botanic Garden, Mount Annan, NSW 2567, Australia; Centre for Ecosystem Science, School of Biological, Earth and Environmental Sciences, Sydney NSW 2052, Australia; The Australian PlantBank, Australian Institute of Botanical Science, Australian Botanic Garden, Mount Annan, NSW 2567, Australia; Centre for Ecosystem Science, School of Biological, Earth and Environmental Sciences, Sydney NSW 2052, Australia; The Australian PlantBank, Australian Institute of Botanical Science, Australian Botanic Garden, Mount Annan, NSW 2567, Australia; The Australian PlantBank, Australian Institute of Botanical Science, Australian Botanic Garden, Mount Annan, NSW 2567, Australia; The Australian PlantBank, Australian Institute of Botanical Science, Australian Botanic Garden, Mount Annan, NSW 2567, Australia; The Australian PlantBank, Australian Institute of Botanical Science, Australian Botanic Garden, Mount Annan, NSW 2567, Australia

**Keywords:** *Alectryon subdentatus*, *Callitris baileyi*, climate change, generalized additive models, germination niche, seed testing, temperature, *Wollemia nobilis*

## Abstract

**Background and Aims:**

Seed germination is strongly influenced by environmental temperatures. With global temperatures predicted to rise, the timing of germination for thousands of plant species could change, leading to potential decreases in fitness and ecosystem-wide impacts. The thermogradient plate (TGP) is a powerful but underutilized research tool that tests germination under a broad range of constant and alternating temperatures, giving researchers the ability to predict germination characteristics using current and future climates. Previously, limitations surrounding experimental design and data analysis methods have discouraged its use in seed biology research.

**Methods:**

Here, we have developed a freely available R script that uses TGP data to analyse seed germination responses to temperature. We illustrate this analysis framework using three example species: *Wollemia nobilis*, *Callitris baileyi* and *Alectryon subdentatus*. The script generates >40 germination indices including germination rates and final germination across each cell of the TGP. These indices are then used to populate generalized additive models and predict germination under current and future monthly maximum and minimum temperatures anywhere on the globe.

**Key Results:**

In our study species, modelled data were highly correlated with observed data, allowing confident predictions of monthly germination patterns for current and future climates. *Wollemia nobilis* germinated across a broad range of temperatures and was relatively unaffected by predicted future temperatures. In contrast, *C. baileyi* and *A. subdentatus* showed strong seasonal temperature responses, and the timing for peak germination was predicted to shift seasonally under future temperatures.

**Conclusions:**

Our experimental workflow is a leap forward in the analysis of TGP experiments, increasing its many potential benefits, thereby improving research predictions and providing substantial information to inform management and conservation of plant species globally.

## INTRODUCTION

Seed germination is a critical and sensitive stage of the plant life cycle (Grubb, 1977) and temperature is a key driver catalysing the germination process (Probert, 2000; Donohue, 2002; Walck *et al.*, 2011; Cochrane, 2019). Seeds of a given species have an ‘optimal’ temperature window wherein the highest and most rapid germination occurs; outside of this range, the germinability of seeds can dramatically decline (Cochrane, 2020). With average global temperatures predicted to increase by at least 1.5 °C by 2100 (IPPC, 2018), the timing and success of germination for many species could be impacted (Parmesan and Hanley, 2015). Changes to the window of conditions conducive for germination can affect subsequent life history traits that are important for plant survival such as timing of reproduction and size at reproductive maturity (Donohue, 2002). These can translate into ecosystem-wide effects, such as changes to food availability, soil stability and fuel loads. Understanding how the germination of a species responds to climate is a crucial step in the management and conservation of that species, but these data are often difficult to obtain. For a comprehensive understanding of a species’ germination niche, one could observe emergence *in situ* with seasonal field trips (Lloret *et al.*, 2004; Tormo *et al.*, 2014); however, this approach is costly, time consuming and otherwise impractical without long-term commitment. Less comprehensive, but similar, information can be obtained more rapidly and economically under controlled laboratory conditions using a bidirectional thermogradient plate (TGP).

**Fig. 1.**
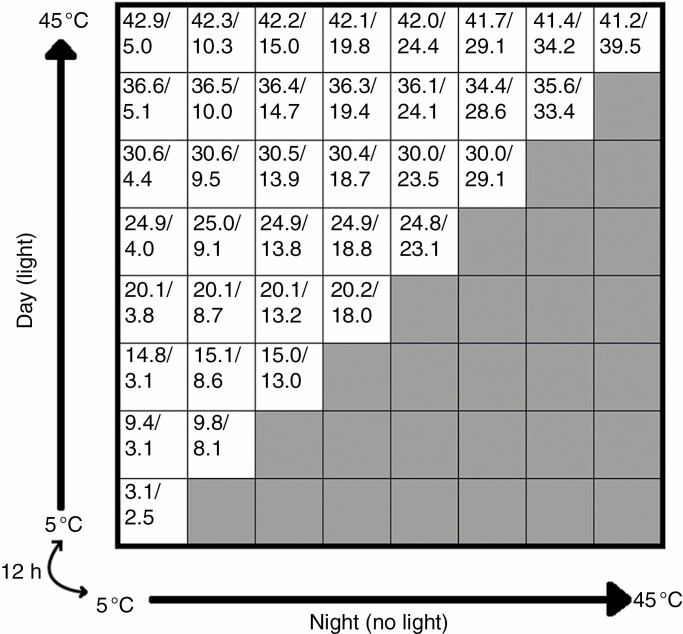
Example set-up of the thermogradient plate for seed germination studies. The axes are the thermogradients which swap over every 12 h to simulate ‘day’ and ‘night’ temperatures. These thermogradients are set to 5 °C on the cold side and 45 °C on the warm side. The numbers inside the cells represent average day/night temperatures (°C) that each cell experienced during the *Alectryon subdentatus* experiment. The shaded cells represent cells that are not typically used for ecological studies, as they have higher night-time than day-time temperatures. In this set-up (36 used cells), each cell is 95 mm across.

Thermogradient plates have been used in seed germination laboratory experiments since the late 1950s (Elliott and French, 1959; Larsen and Skaggs, 1969). Essentially, the equipment consists of an aluminium plate which is cooled along one edge and heated along the perpendicular edge, creating a two-way temperature gradient. The plate is typically divided into even gridded cells, where the among-cell temperature differences are roughly consistent. Seeds generally experience diurnal temperature differences in the wild, and these should be incorporated during seed germination experiments (Baskin and Baskin, 2014). A bidirectional TGP enables the direction of the thermogradient as well as the light to be changed at set time periods, so each cell has a ‘day’ and ‘night’ simulated temperature (Fig. 1). The TGP is a powerful research tool for linking seed germination with the temperature range of the source environment, as seeds can be tested at a user-determined temperature range (typically between 5 and 40 °C). This makes it well suited for climate-based studies because, for most climates, seed germination can be tested at current and future temperatures, giving researchers the ability to make more accurate predictions of climate change impacts.

Seed biologists have been hesitant to use TGPs in their research due to several key limitations. Notably, multiple runs on the TGP are required to produce replication similar to traditional incubator-based experiments, which may be impractical to achieve and can lead to further statistical problems. To overcome this, the individual seed is usually used as the unit of replication for TGP experiments (Cochrane, 2020), but this method can complicate statistical analysis. Additionally, germination *in situ* is affected by extrinsic factors other than temperature, such as moisture availability and soil microbe relationships, and intrinsic factors such as seed dormancy, all of which need to be considered when designing an *ex situ* germination experiment. The experimental design will also be influenced by available lab equipment, the amount of seed available and research hypotheses.

Thermogradient plates have previously been used to link species’ germination to climate. Fernández-Pascual *et al.* (2015) used a TGP to germinate two provenances (one montane and one sub-alpine) of *Carex diandra* (Cyperaceae), a widely distributed northern hemisphere sedge. They found that alternating temperatures were important for germination, and higher temperatures were likely to increase germination overall; however, shifts in germination time affected the provenances differently. In a comprehensive study, Cochrane (2020) used a TGP to germinate 102 species from southern Western Australia, an area with Mediterranean (winter) rainfall. The author then examined the relationship between the TGP germination data and local seasonal climate variables, and reported that most species could germinate at temperatures higher than they are currently experiencing during the wettest months, meaning they were likely to be well within their thermal tolerance for germination. However, some species were found to proabaly undergo a range shift where future local temperatures were predicted to exceed their optimum. In another study, Catelotti *et al.* (2020) used the TPG to germinate six *Persoonia* species from south-eastern Australia. They found that each species had unique temperature sensitivity and optima, but reported that germination probability was likely to decrease under predicted future climate scenarios. While these studies illustrate the TGP as a powerful research tool, they utilized very different designs and methods of analysis. These differences among experiments stem from an absence of practical guidelines for generating and analysing TGP data, and can render it difficult to make direct comparisons among studies.

Here, using three Australian endemic plant species as case studies, we aim to provide an accessible statistical framework describing how data generated from a TGP with the seed as a unit of replication can be modelled and predicted into current and future temperatures using the open source software R. Our methodology is globally relevant, and the data generated can be applied to on-ground restoration or conservation management projects, as well as informing ecological research and applied horticulture, giving insight into current and potential future germination patterns of plant species.

## MATERIALS AND METHODS

### Case study species

*Alectryon subdentatus* (F. -Muell. Ex Benth.) Radlk. (Sapindaceae) is an erect shrub to small tree to 11 m tall, and is distributed from central east Queensland to north-east New South Wales (NSW). *Alectryon subdentatus* occurs in vine thickets and dry rainforest communities often on steep rocky slopes in protected gullies (Edwards and Gadek, 2001). Flowering typically occurs in spring and summer (September to February) following enough rainfall. The fruits are hard two-lobed capsules with 1–2 seeds, each almost entirely covered by a red aril, and seed dispersal typically occurs in summer. *Alectryon subdentatus* is also capable of resprouting from the base of the main stem.

*Callitris baileyi* C.T.White (Cupressaceae) is a slender tree up to 18 m, distributed from south-east Queensland to far-north NSW. It is found in Eucalypt woodland on rocky, hilly or mountainous areas, usually near creeks, on shallow, often clay soils. The species is listed as Endangered under the NSW Biodiversity and Conservation (BC) Act 2016. Fruiting has been recorded all year round, and male and female cones occur on the same tree. Through field observations, it appears that the cones are weakly serotinous, with most seeds being dispersed during summer. The species is thought to be an obligate seeder, but its capacity to resprout vegetatively has not been determined (Denham *et al.*, 2016).

*Wollemia nobilis* W.G.Jones, K.D.Hill & J.M.Allen (Araucariaceae) is a tall tree (to 40 m) growing in warm temperate rain forest in a remote sheltered gorge in Wollemi National Park on the Central Tablelands of NSW. The species is restricted to a several stands of <100 adult trees, and a bank of seedlings on the floor of the gorge. Consequently, it is listed as Critically Endangered under the NSW BC Act 2016 and the federal Environment Protection and Biodiversity Conservation Act 1999. Cones mature in mid to late summer and seeds are shed from the disintegrating cones for some months after. Seeds are free from the cone scales and are approx. 8–10 × 6–8 mm in length × width (Offord *et al.*, 1999; Offord and Meagher, 2001). The species also regenerates by resprouting from the base of the tree and may form a large clump of stems, each of which can develop into a large trunk in high light.

### Experimental design

Experiments were conducted in October 2017 for *W. nobilis*, January 2020 for *C. baileyi* and February 2021 for *A. subdentatus*. Notably, each species was set up differently on the TGP, which provided the stimulus for this study. For all species, only cells that received 12 h of light during the warm part of the diurnal cycle were used. For *W. nobilis*, the TGP was set up in a 21-cell half grid (six cells along the *x*- and *y*-axis) with large cell sizes to fit five replicate 43 mm polycarbonate sample jars, each with ten seeds. The jars were arranged with a central jar and four around the outside, and were rotated weekly within their cells so every jar experienced, on average, the same temperatures within the cell. We modified this set-up for data analysis by combining the replicate jars so that each cell was represented by 50 seeds. For *C. baileyi*, the TGP was set up as a 45-cell half grid (nine cells along the *x*- and *y*-axis), each with one 70 mm Petri dish containing ten seeds. Finally, for *A. subdentatus*, the TGP was set up in a 36-cell half grid (eight cells along the *x*- and *y*-axis) with ten seeds per cell. This species was run concurrently with another species using 90 mm polycarbonate bi-plates (Livingstone International, Australia), in which the two species were separated from each other by a built-in barrier, and the plates were rotated a random amount every 3–4 d. Germination was checked every 1–2 weeks for *W. nobilis* and *C. baileyi*, and every 3–4 d for *A. subdentatus*, and was scored when the radicle emerged > 1 mm from the seed. Viability was calculated by cut test (Ooi *et al.*, 2004) as a proportion of the seed lot before the experiment, as extreme temperatures on the TGP can affect viability differently from mild temperatures. This proportion was then factored into the data. The TGP cell temperatures were recorded using an infrared thermometer (ZyTemp TN408LC, Bacto Laboratories Pty Ltd, Mount Pritchard, NSW) on the surface of the plate, during both light and dark periods, on days on which germination was checked. Final cell temperatures were produced by averaging temperature readings over the experimental period. All seeds were sown onto Petri dishes or jars that were half filled with water agar (0.07 % agar; depth 5–10 mm).

### Climate models

We focused on air temperature as the climate variable of interest in our workflow. It is possible to model soil temperature (see Kearney *et al.*, 2020); however, predictions of soil temperature become more uncertain in future climate predictions due to a range of possible feedback mechanisms. Furthermore, seeds can be located at various depths from the surface, making predicting the exact temperature the seeds will experience in the soil impractical.

At the time of this study, current temperature predictions were modelled with WorldClim 2.1 and downloaded from Worldclim (worldclim.org/data/worldclim21.html; accessed May 2021). In this dataset, monthly mean maximum and minimum temperatures (*t*_max_ and *t*_min_) were collected from approx. 17 000 weather stations and then spatially interpolated at a 1 km^2^ resolution for the years 1970–2000. The interpolated temperatures displayed high correlation (>99 %) with observed temperature values globally (Fick and Hijmans, 2017).

Future climate predictions were based on predicted *t*_max_ and *t*_min_ produced from Coupled Model Intercomparison Project Phase 6 (CMIP6) models and were downloaded from Worldclim (https://worldclim.org/data/cmip6/cmip6_clim2.5m.html; accessed May 2021). The models include a range of shared socio-economic pathways (SSPs) to predict temperatures. Since each SSP considers different political and environmental futures and models 43 greenhouse gases at a monthly resolution, climate models can be easily modified to incorporate a range of scenarios. For further information regarding SSPs, see Meinshausen *et al.* (2020). We used SSP5-8.5 representing the ‘fossil-fuel development’, leading to a global average temperature increase of approx. 5 °C. This scenario was chosen as the actual temperature increase could be anywhere between this and the ‘sustainability0’ SSP1-2.6 scenario. We included all eight available CMIP6 SSP5-8.5 model variations from the Worldclim database for the years 2081–2100 at a 2.5 min (approx. 4.5 km^2^) spatial resolution and averaged for each monthly data point.

### Data analysis

For all analyses, we used R (v4.2; R Core Team, 2020). We used an R notebook to produce a repeatable, easy to understand data analysis workflow. This flexible method helps guide users through several decisions in an already globally used environment and is available at https://zenodo.org/record/5457222. Each species was run and analysed separately using this workflow.

For a comprehensive understanding of the germination dynamics across the TGP, >45 germination indices were calculated using the ‘germination.indices’ function within the {germinationmetrics} package (Aravind *et al.*, 2019). To use this function with irregular germination scoring intervals, we built a function that generated consistent germination intervals by interpolating data for non-checked days using a generalized additive model (GAM). Importantly, this can be adapted to suit any germination experiment, and should increase the utility of ‘germination indices’. The raw data were visualized using an interpolated rasterized quilt plot with day temperature as the *x*-axis, night temperature as the *y*-axis and a chosen germination index as the *z*-axis. Here, we only report on final germination proportion, and provide additional model outputs for time to 50 % germination (*t*_50_) as Supplementary Information.

To test the relationship between observed germination and temperature treatments, GAMs were used because they consider both linear and non-linear relationships. The ability to handle complex non-linear relationships was important since we expected *a priori* that there would be non-linear relationships between germination and temperature. To increase user confidence, an automated model selection framework was included to test multiple plausible model specifications, including linear terms, single smooth terms as well as more complex basis functions such as bivariate smooths and tensor products. The GAMs were fitted using the {mgcv} package (v1.8; Wood, 2006); the specific models fitted for each species can be seen in the code provided, and more detailed information on how to fit these model types in R can be found in Wood (2017).

To evaluate model outputs and choose a single model to proceed with analysis for each species, we used an approach based on testing predictive performance. Specifically, we compared three outputs: (1) the root mean square prediction error (RMSE) for models fitted on all the data; (2) the Pearson’s correlation between the predicted and fitted values for models fitted on all the data; and (3) the prediction error into hold-out samples via Monte Carlo resampling. The resampling procedure implemented involved fitting the models to 90 % of the data and calculating the prediction error into the held-out 10 %, which was repeated 100 times for each model tested. This number should be tested with different values (i.e. sensitivity analysis) and selected when the mean error stabilizes. To simplify model selection, all model outputs were placed into a table for easy comparison. The resampling distribution also provided a convenient non-parametric approach for quantifying the mean error and confidence interval (95 % percentile of distribution) for the model chosen to continue analysis for each species. The modelled data were then visualized as a quilt plot and compared with the raw data plot. Here, we modelled final germination proportion for each species using binomial GAMs.

Next, a decimal latitude and longitude were inputted. This was typically the seed source location, or a generalized location of interest (such as the vegetation community or reserve). The locations for *A. subdentatus* and *C. baileyi* were Deriah Aboriginal Area in the NSW north-west slopes region and Mallanganee in the NSW north coast region, respectively. Due to rarity in the wild, *W. nobilis* seeds were collected from a cultivated source (National Aboretum, Canberra) and we inputted a generalized location in Wollemi National Park, NSW due to the sensitivity around the location of the wild population. For each species location and monthly *t*_min_ and *t*_max_ values were extracted from the downloaded models, and final germination proportion was predicted for both current and future temperatures using the selected GAM.

## RESULTS

**Fig. 2.**
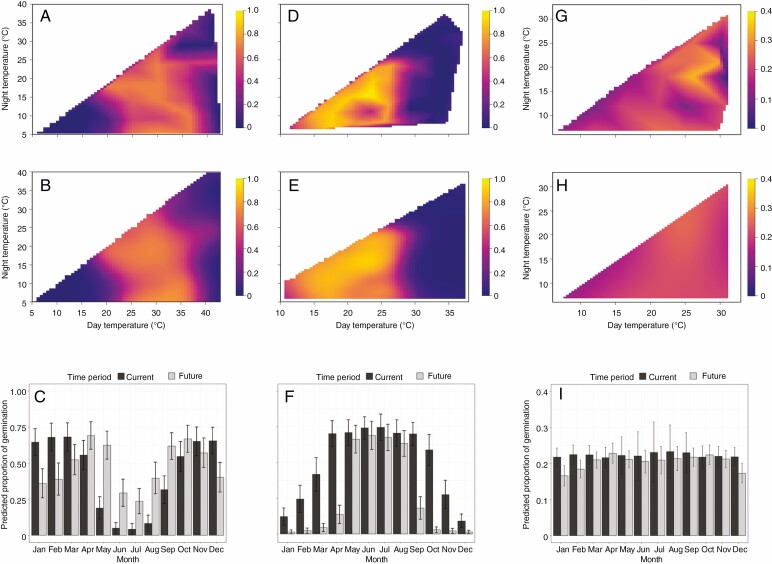
Observed and modelled germination data for three case study species under multiple temperature regimes: (A–C) *Alectryon subdentatus*; (D–F) *Callitris baileyi*; (G–I) *Wollemia nobilis*. Observations were generated using a TGP, and modelled using generalized additive modelling. The first row (A, D and G) shows rasterized quilt plots of the observed data, with day temperature (°C) on the *x*-axis, night temperature on the *y*-axis and final germination proportion on the *z*-axis. The second row (B, E and H) shows the same plots, but with the modelled data rather than the observed data. These plots show the best fitting model for each species. This is a tool to visualize model fit. The final row (C, F and I) shows the final germination proportion predicted into real world current and future predicted temperature for each month at the location of the source population for each species (except for *W. nobilis*). Current temperature predictions are downloaded from Worldclim models. The future temperatures predictions are an average (± s.e.) temperature from eight CMIP6 models under the IPPC shared socioeconomic pathway SSP5-8.5, for the years 2081–2100.

The final germination proportion was high (≥ 0.8) for *Alectryon subdentatus* and *Callitris baileyi*. In contrast, the final germination proportion was low for *Wollemia nobilis*, with a maximum of 0.34. *Alectryon subdentatus* had a reasonably high but constrained temperature window for germination, with a preference for fluctuating rather than constant temperatures. The final germination proportion was ≥ 0.5 when day temperatures were between 25 and 35 °C; however, germination gradually decreased to < 0.1 as day and night temperatures surpassed 35 and 30 °C, respectively, or were below 25 and 20 °C, respectively (Fig. 2A). The optimal temperature for maximum germination of this species (0.8) occurred at 30/14 °C. In contrast, *C. baileyi* showed a restricted and cool temperature window with little variation in diurnal temperatures. Germination occurred at temperatures ranging from 6 to 25 °C, and was considerably reduced or inhibited above these temperatures (Fig. 2D). The maximum proportion of germination (1.0) for this species occurred at 23/15 °C. Finally, *W. nobilis* had a very broad temperature window and germinated uniformly over almost all temperature combinations (Fig. 2G). Consequently, there was little effect of temperature on its final germination.

The best performing models that fit our raw germination data are outlined in Table 1. The extent to which the selected model for each species fit the observed data was good (Fig. 2), with mean error and RMSE values ranging from 0.09 to 0.13 and from 0.066 to 0.083, respectively, and correlations between the observed and predicted data ranging from 0.59 to 0.98. *Wollemia nobilis* had the poorest fit due to its broad temperature window. The contrasting temperature windows for germination for all species were further illustrated when the predicted data were modelled under current *in situ* temperatures. *Alectryon subdentatus* germination was predicted to primarily occur during warmer months in spring, summer and autumn (October–March; Fig. 2C), whereas maximum germination for *C. baileyi* was predicted to occur in autumn, winter and spring (April–October; Fig. 2F). *Wollemi nobilis* was predicted to germinate consistently across the whole year (Fig. 2I). When predicted germination was forecast under future climate, there were considerable seasonal changes to germination for *A. subdentatus* and *C. baileyi* while little change was predicted for *W. nobilis*.

**Table 1.** Output of an R script used to model final germination proportions for the three study species using data generated from germination experiments on a thermogradient plate

Species	Model	Mean error	Lower 95 %	Upper 95 %	RMSE	Correlation
*A. subdentatus*	prop_germ ~ te(day_temp, night_temp, bs = ‘cr’)	0.128	0.035	0.242	0.126	0.906
	prop_germ ~ te(day_temp, night_temp, bs = ‘tp’)	0.134	0.043	0.255	0.130	0.900
	**prop_germ ~ s(day_temp, night_temp, bs = ‘tp’)**	**0.134**	**0.022**	**0.235**	**0.083**	**0.963**
	prop_germ ~ s(day_temp, bs = ‘tp’) + s(night_temp, bs = ‘tp’)	0.141	0.037	0.278	0.129	0.901
	prop_germ ~ te(day_temp, night_temp, bs = ‘ts’)	0.144	0.039	0.252	0.132	0.897
	prop_germ ~ ti(day_temp, night_temp, bs = ‘tp’)	0.25	0.074	0.521	0.244	0.569
*C. baileyi*	**prop_germ ~ s(day_temp, night_temp, bs = ‘tp’)**	**0.116**	**0.036**	**0.226**	**0.082**	**0.976**
	prop_germ ~ s(day_temp, bs = ‘tp’) + s(night_temp, bs = ‘tp’)	0.129	0.047	0.251	0.111	0.955
	prop_germ ~ te(day_temp, night_temp, bs = ‘tp’)	0.131	0.037	0.244	0.110	0.956
	prop_germ ~ te(day_temp, night_temp, bs = ‘ts’)	0.134	0.045	0.253	0.124	0.943
	prop_germ ~ te(day_temp, night_temp, bs = ‘cr’)	0.137	0.035	0.238	0.113	0.953
	prop_germ ~ ti(day_temp, night_temp, bs = ‘tp’)	0.28	0.097	0.505	0.240	0.767
*W. nobilis*	prop_germ ~ te(day_temp, night_temp, bs = ‘ts’, k = 4)	0.073	0.023	0.153	0.073	0.484
	prop_germ ~ s(day_temp, night_temp, bs = ‘tp’, k = 4)	0.076	0.028	0.146	0.078	0.232
	prop_germ ~ s(day_temp, bs = ‘tp’, k = 4) + s(night_temp, bs = ‘tp’, k = 4)	0.077	0.03	0.165	0.070	0.491
	prop_germ ~ ti(day_temp, night_temp, bs = ‘tp’, k = 4)	0.081	0.015	0.17	0.074	0.421
	**prop_germ ~ te(day_temp, night_temp, bs = ‘tp’, k = 4)**	**0.09**	**0.027**	**0.258**	**0.066**	**0.598**
	prop_germ ~ te(day_temp, night_temp, bs = ‘cr’, k = 4)	0.09	0.024	0.188	0.066	0.595

Mean error is gained by hold-out samples via Monte Carlo resampling. Ninety per cent of the data were modelled and predicted into the held-out 10 % for 100 iterations. Lower and upper 95 % use the mean error to create confidence intervals (95-percentile of distribution). RMSE is the root mean squared error, a common measure of model error. Correlation is the Person’s correlation between the modelled and observed data. The model in bold was that which was selected as the best fitting and was used to make subsequent predictions and plots. Prop_germ refers to the final germination expressed as a proportion. The letters before the model terms refer to the smoother that was used in the model. ‘te’ and ti’ are tenser terms, while ‘s’ is a smooth term. The letters after ‘bs’ within the model refer to the type of smooth term used. ‘cr’ refers to a cubic spline, ‘tp’ refers to a thin plate smoother and ‘ts’ is also a thin plate smoother, with a modification to the smoothing penalty, so null space is penalized. ‘K’ refers to the number of knots used and is modified when there are smaller amounts of data.

## DISCUSSION

### Germination and climate modelling

Final germination was high overall for *A. subdentatus* and *C. baileyi*, and was lower for *W. nobilis*, although this was typical of the latter species (Offord and Meagher, 2001). In general, a range of ungerminated but apparently viable seeds remained for all species, which suggested that some temperatures may have inhibited germination. For all three species, the data generated using a TGP can give key insights into current and future ecology. For example, a seasonal shift was predicted for *A. subdentatus* with declines in germination in summer and early autumn (December–March; Fig. 2C) and an increase during milder months between late autumn and early spring (May–September; Fig. 2F). This shift could have substantial impacts on *A. subdentatus* recruitment, as rainfall in the region predominantly occurs in the summer months, a pattern that is predicted to intensify with climate change (Ekström *et al.*, 2015). Furthermore, soil moisture is predicted to decrease in winter and spring due to a reduction in rainfall during these seasons (Ekström *et al.*, 2015). *Callitris baileyi* was predicted to experience an overall decline in its maximum germination and a reduced germination period occurring over 4 months in late autumn and winter (May–August; Fig. 2I). When coupled with changes to the moisture regimes outlined above, this may also lead to a decrease in overall recruitment for *C. baileyi* which could have flow-on consequences for the population in the future. While *W. nobilis* did not show a strong response to temperature, climate change-related factors other than temperature for germination are predicted to be greater drivers of species loss (Offord, 2011; Zimmer *et al.*, 2015).

### The use of a thermogradient plate

While the TGP links germination to temperature in a way that provides unique predictions of the possible impacts of climate change, it must be noted that these predictions are made without considering key extrinsic factors such as moisture, soil microbes, weather, disturbance and other abiotic and biotic factors that may interact to influence germination; such complexity is unlikely to be replicated in a laboratory. Other limiting factors in the germination process, such as the presence or absence of seed dormancy, must also be considered when running a TGP experiment.

Seed dormancy is present in many species and is particularly prominent in habitats with high levels of disturbance (Pausas and Keeley, 2014; Dayrell *et al.*, 2017; Collette and Ooi, 2021). Before conducting *ex situ* germination experiments on such species, specific treatments may be required to alleviate dormancy. For a TGP experiment, this can be problematic, but can be overcome if the dormancy type is known. For example, if the species has known physical dormancy, all seeds can be scarified to break dormancy before sowing seeds on the TGP. Species with a physiological component of dormancy are more complicated, as they may require a specific sequence of seasonal temperatures for dormancy break to occur, and then may require other factors such as smoke, heat and light to promote germination (Thompson and Ooi, 2010, 2013). Furthermore, these species may take a long time to germinate due to a requirement for after-ripening (Baskin and Baskin, 2014) which may account for the low germination results for *W. nobilis* (Offord and Meagher, 2001). In addition to physiological dormancy, species with a morphological component may take a long time to germinate (Phartyal *et al*., 2012). For such species, it is advisable to first determine which treatments induce or increase germination via traditional experimentation, and then apply those treatments to all seeds before placing them on the TGP. For example, the species might need a smoke treatment to promote germination (Baskin and Baskin, 2014; Mackenzie *et al.*, 2016; Collette and Ooi, 2020) which could be applied to all seeds via smoke water agar (Emery and Lacey, 2010). To test the interactive effects of smoke and temperature, two runs would need to be completed on the TGP, one with and one without smoke. Multiple TGP runs could then be treated as a blocking factor in statistical models.

The method described here used the seed as a unit of replication, which has been done previously (Cochrane, 2020). This remains an inherent risk with using a TGP. Without conducting multiple runs on a TGP with the same species and seed batch, it is impossible to identify how much variation can be attributed to the lack of replication of Petri dish plates. Even with subsequent runs on a TGP, differences in conditions and ripening of seeds will affect the results. This could be dealt with by using temporal blocking in the statistical model; however, this approach contradicts the purpose of our method, which is to produce more detailed and extrapolated information from a single TGP run than previously possible. Our framework centres on fitting a non-linear curve between germination and temperature, and predicting the response of the former from the latter, rather than analysing for statistical difference among temperature cells. However, we advise that care should be taken when interpreting results from the method described. If seeds are available after a TGP run, much more detailed statistical insights can be gained from using traditional incubator-based experiments.

With these limitations, when is it beneficial to use TGPs rather than incubators? Firstly, if the research questions revolve around how climate may influence germination, the TGP is the preferred option, as it covers a large range of alternating and constant temperatures which are not practical to achieve with incubators. For example, to examine temperature effects on germination of alpine seeds with incubators, Sommerville *et al.* (2013) used six incubators running at a mix of constant and fluctuating temperatures. Commander *et al.* (2017) also used six incubators, starting from constant 10 °C and increasing to 35 °C in 5 °C increments. Even with the use of six incubators, the range of temperatures covered in these studies is well below the temperature range and variation (fluctuating temperatures) achievable on a TGP. While using a large temperature range on the TGP, a temperature gradient can be introduced with the Petri dish. This can be nullified by rotating the dish at evenly spaced intervals (unpubl. data). The large range of temperatures that a TGP can cover allows researchers to more feasibly study both current and future predicted temperatures. Secondly, with limited seeds available, using the method described herein generates a large amount of information for a relatively small number of seeds. For example, the studies described above using multiple incubators required between 240 and 750 seeds, depending on available seed per species (Sommerville *et al.*, 2013; Commander *et al.*, 2017). By comparison, the TGP method used here can be completed with as few as 360 seeds per species while covering a broader temperature range. The use of fewer seeds is also advantageous for rare and threatened species that may produce few or no viable seeds in a season (Cochrane *et al.*, 2001). Finally, if a laboratory is already equipped with a TGP but seed biologists are hesitant to use it, our method provides a pathway to confidently use data generated to gain meaningful results.

### Conclusions

The dynamic relationship between temperature and seed germination under the developing threat of climate change is a key management issue for plants globally. In this study. we have demonstrated one method to explore this relationship and predict the germination niche for a species under current and future temperatures. These data can then be considered when determining and prioritizing species management actions. More broadly, this method could also be used for understanding how to propagate species from seeds for horticulture or conservation purposes. For our case study species, shifts in future climate could lead to a variety of outcomes, including range shifts, changes in phenology leading to ecosystem-wide effects, and potential changes to plant fitness (Parmesan and Hanley, 2015). The workflow described here builds on the growing body of literature examining temperature effects on germination by guiding researchers on effective and efficient analysis of TGP data.

## Supplementary Material

mcac026_suppl_Supplementary_File_1

## Data Availability

The data that support the findings of this study are openly available in Zenodo at https://doi.org/10.5281/zenodo.5457222.
