# Supplementary material for: Stepping up to the thermogradient plate: a data framework for predicting seed germination under climate change
Source: Ann Bot. 2022 Feb 25;129(7):787–94. doi: 10.1093/aob/mcac026 (PMC9292609; doi:10.1093/aob/mcac026)
Supplement: mcac026_suppl_Supplementary_File_1 [file mcac026_suppl_supplementary_file_1.docx]

Supplementary file 1

***Alectryon subdentatus* t50 analysis**

**
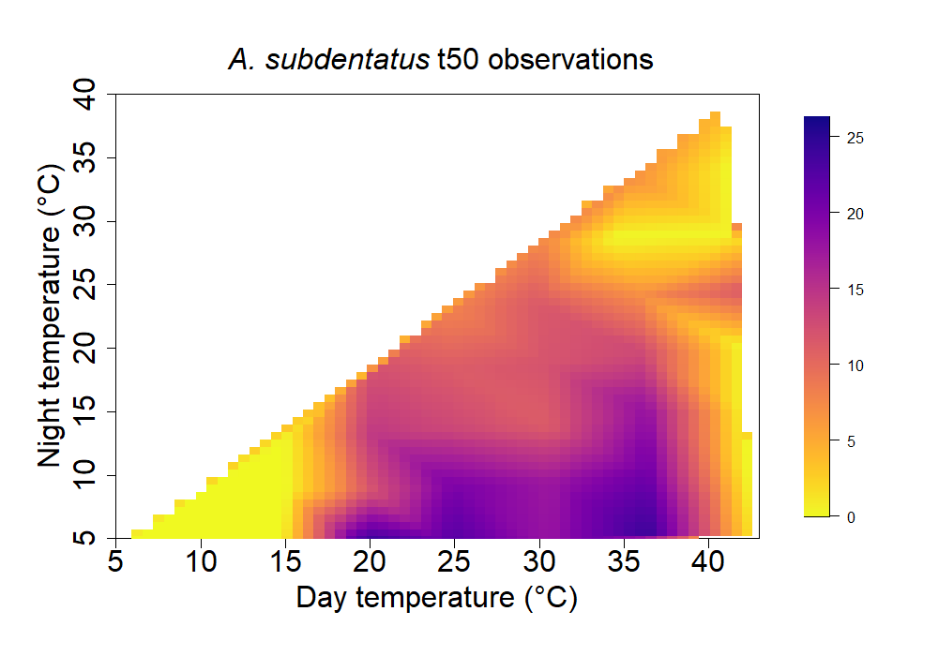
**

1. **Observed t50 (days)**


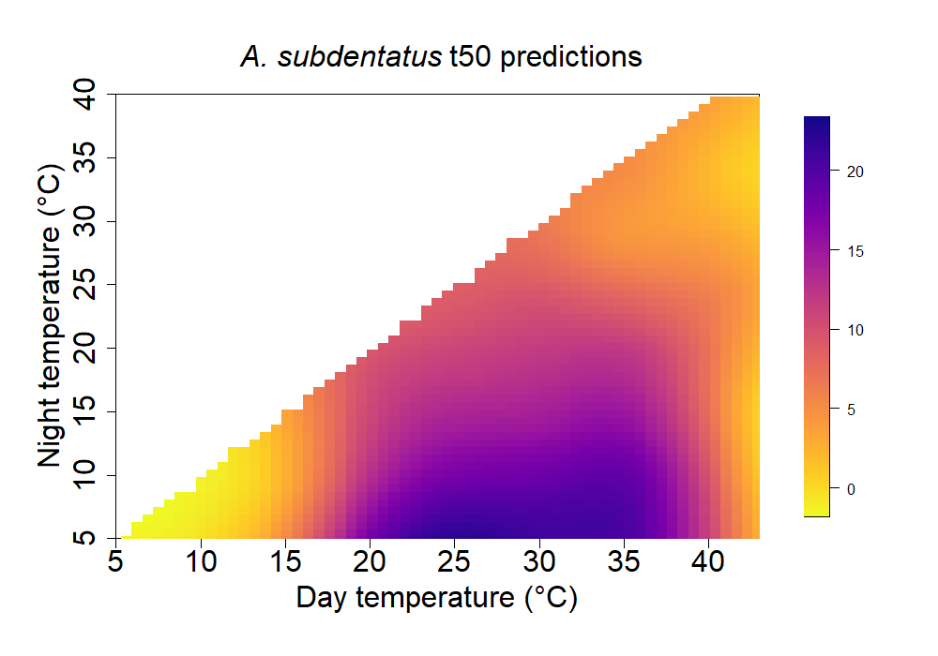


1. **Modelled t50 (days)**

**
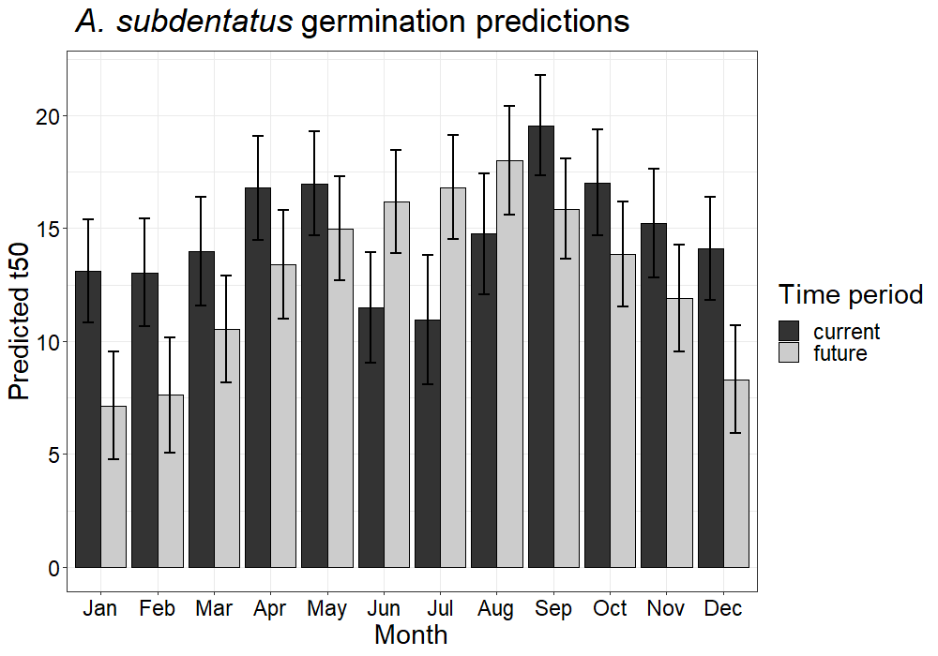
**

1. **Predicted t50 (days) for each month based on current and future climate. Bars represent one standard error**

***Callitris baileyi* t50 analysis**

**
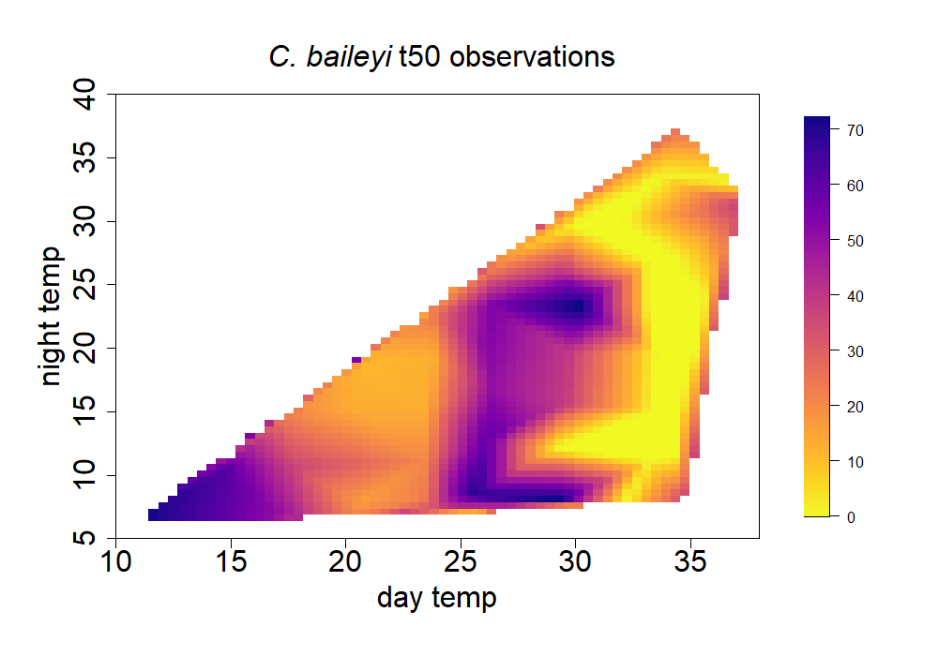
**

1. **Observed t50 (days)**


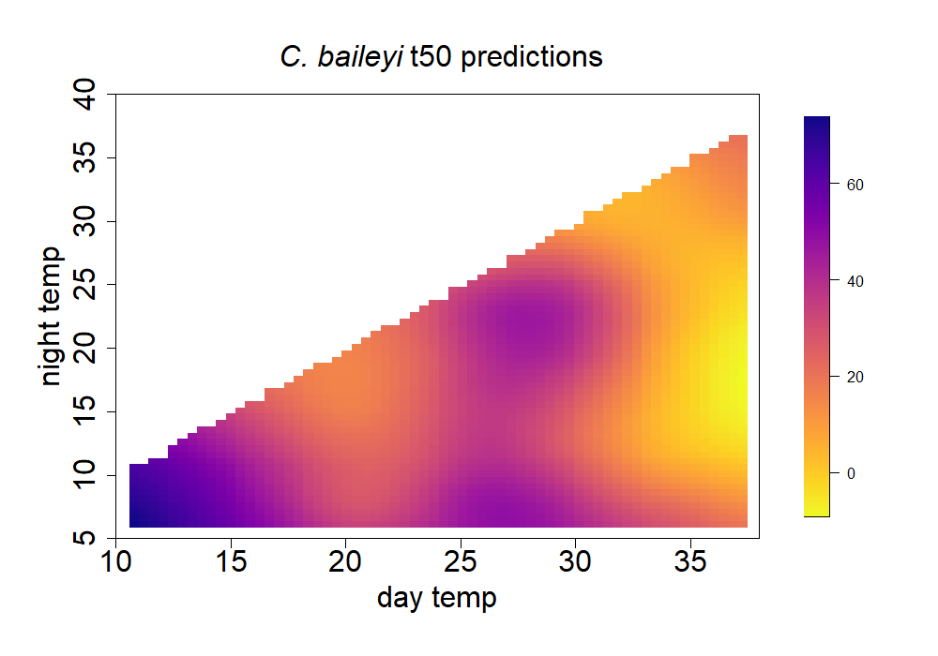


1. **Modelled t50 (days)**

**
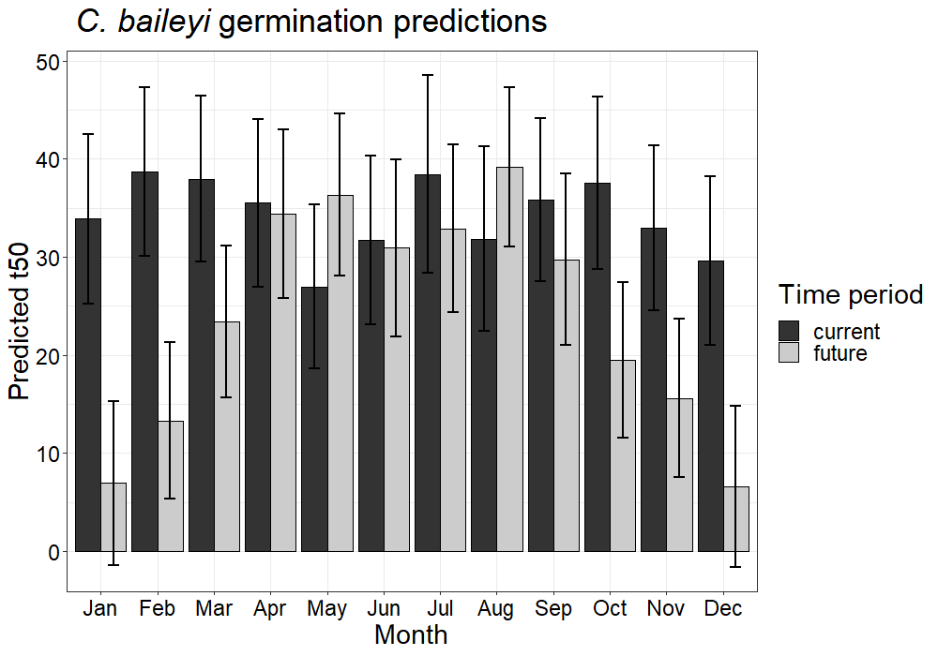
**

1. **Predicted t50 (days) for each month based on current and future climate. Bars represent one standard error.**

**
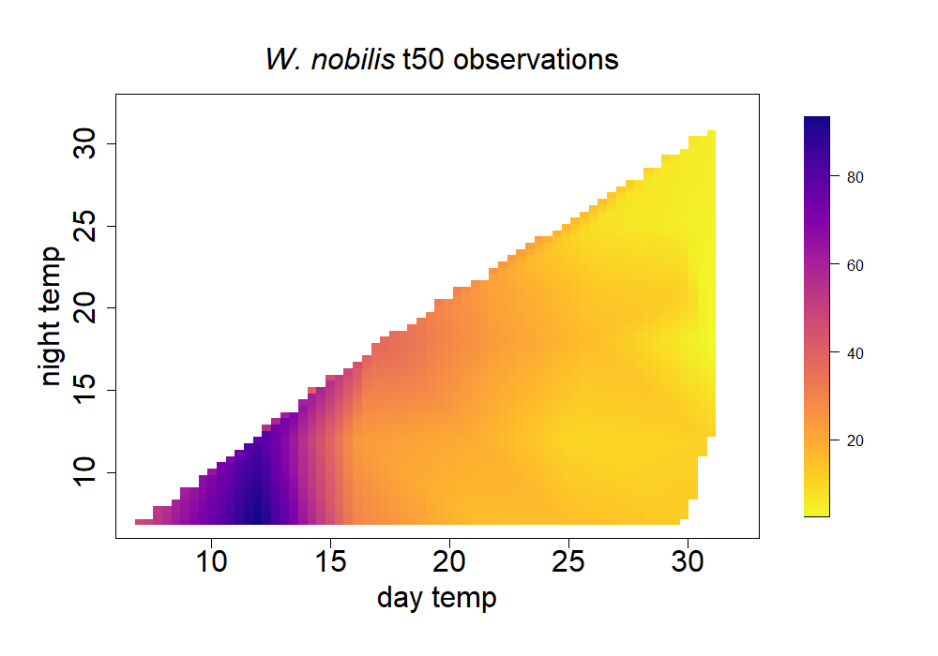
**

1. **Observed t50 (days)
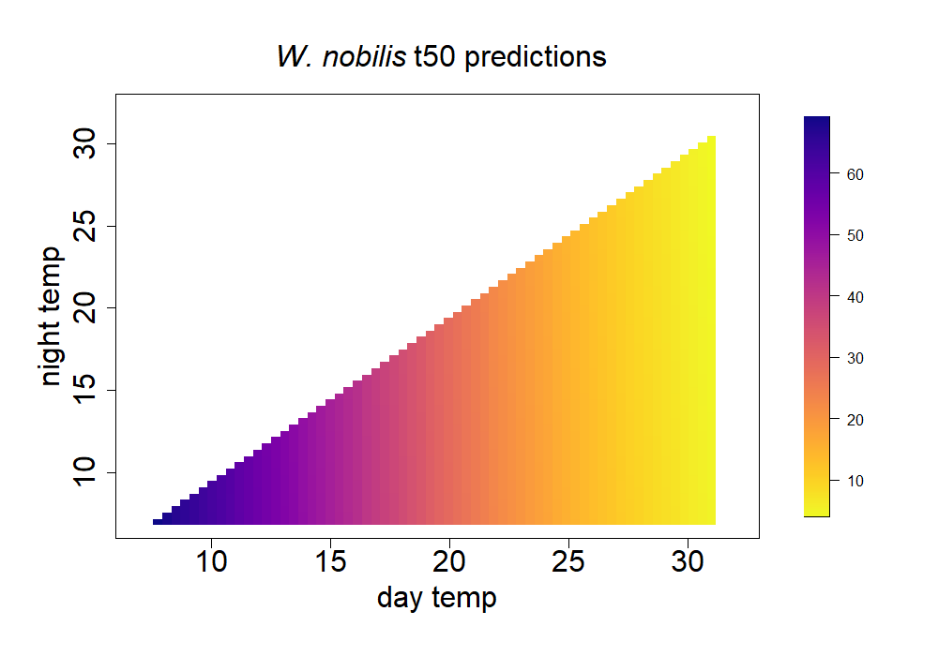
**
2. **Modelled t50 (days)**

**
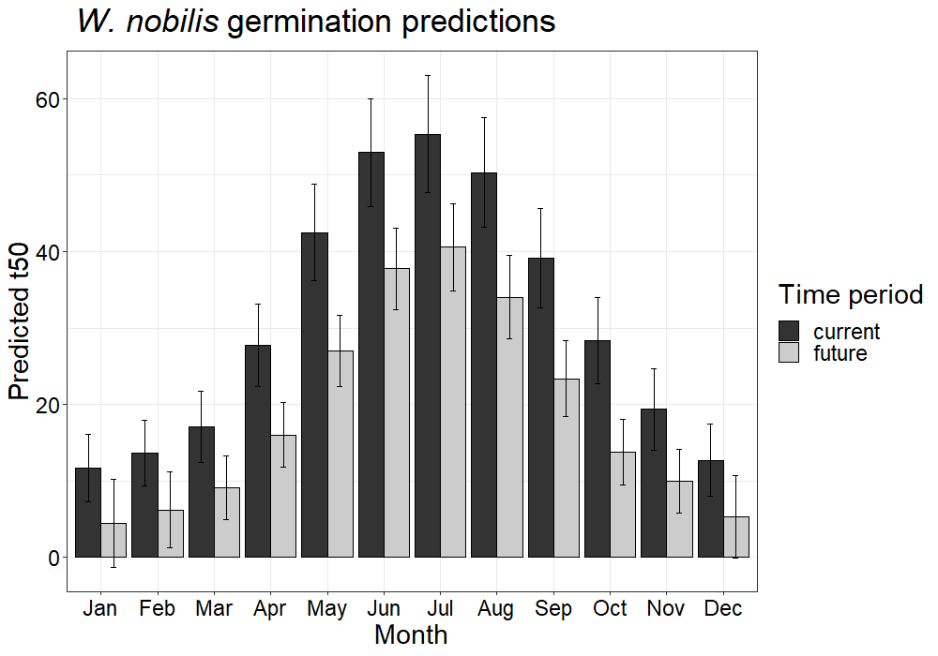
**

1. **Predicted t50 (days) for each month based on current and future climate. Bars represent one standard error.**

**Table. The outputs of all models. The models listed here are the ‘default’ models from the script. Mean error is gained by hold-out samples via Monte-Carlo resampling. 90% of the data was modelled and predicted into the held-out 10% for 100 iterations. Lower and upper 95% use the mean error to create confidence intervals (95% percentile of distribution). RMSE is the Root mean squared error, a common measure of model error, Correlation is the Person’s correlation between the modelled and observed data, and best model is indicated by *. This is the model that was chosen to create the figures.**

| **Species** | **Model** | **mean error** | **lower 95%** | **upper 95%** | **RMSE** | **Correlation** | **Best model** |
| --- | --- | --- | --- | --- | --- | --- | --- |
| A. subdentatus | t50_Farooq ~ s(day_temp, bs = "tp") + s(night_temp, bs = "tp") | 4.033 | 1.2 | 7.663 | 3.495522 | 0.9095159 |  |
|  | t50_Farooq ~ s(day_temp, night_temp, bs = "tp") | 4.979 | 2.392 | 8.756 | 3.228925 | 0.9295871 | * |
|  | t50_Farooq ~ te(day_temp, night_temp, bs = "cr") | 5.047 | 2.072 | 8.842 | 4.483424 | 0.8448224 |  |
|  | t50_Farooq ~ te(day_temp, night_temp, bs = "ts") | 5.11 | 2.132 | 9.435 | 4.576851 | 0.8405153 |  |
|  | t50_Farooq ~ te(day_temp, night_temp, bs = "tp") | 5.19 | 1.831 | 8.928 | 4.520039 | 0.8417983 |  |
|  | t50_Farooq ~ ti(day_temp, night_temp, bs = "tp") | 6.618 | 2.834 | 12.231 | 7.091508 | 0.530761 |  |
|  | t50_Farooq ~ s(day_temp, night_temp, bs = "tp") + s(day_temp) +     s(night_temp) | *NA* | *NA* | *NA* | *NA* | *NA* |  |
| C. baileyi | t50_Farooq ~ s(day_temp, bs = "tp") + s(night_temp, bs = "tp") | 15.03 | 7.011 | 27.857 | 15.4056 | 0.793644 |  |
|  | t50_Farooq ~ te(day_temp, night_temp, bs = "cr") | 17.587 | 8.448 | 33.456 | 16.53763 | 0.7575825 |  |
|  | t50_Farooq ~ s(day_temp, night_temp, bs = "tp") | 19.292 | 10.217 | 31.238 | 14.25888 | 0.8439964 | * |
|  | t50_Farooq ~ te(day_temp, night_temp, bs = "tp") | 19.878 | 12.397 | 30.023 | 18.61544 | 0.6757486 |  |
|  | t50_Farooq ~ te(day_temp, night_temp, bs = "ts") | 20.998 | 12.717 | 30.723 | 19.15659 | 0.66398 |  |
|  | t50_Farooq ~ ti(day_temp, night_temp, bs = "tp") | 22.337 | 13.543 | 37.493 | 23.36647 | 0.3721158 |  |
|  | t50_Farooq ~ s(day_temp, night_temp, bs = "tp") + s(day_temp) +     s(night_temp) | *NA* | *NA* | *NA* | *NA* | *NA* |  |
| W. nobilis | t50_Farooq ~ s(day_temp, bs = "tp", k = 4) + s(night_temp, bs = "tp",     k = 4) | 12.305 | 1.282 | 39.626 | 13.32965 | 0.8278816 | * |
|  | t50_Farooq ~ s(day_temp, night_temp, bs = "tp", k = 4) | 13.83 | 3.096 | 26.736 | 14.14232 | 0.8034498 |  |
|  | t50_Farooq ~ te(day_temp, night_temp, bs = "cr", k = 4) | 14.599 | 2.194 | 43.418 | 14.09553 | 0.8049057 |  |
|  | t50_Farooq ~ te(day_temp, night_temp, bs = "tp", k = 4) | 16.754 | 2.2 | 47.222 | 13.08362 | 0.834971 |  |
|  | t50_Farooq ~ te(day_temp, night_temp, bs = "ts", k = 4) | 18.542 | 2.273 | 48.959 | 14.15117 | 0.8073297 |  |
|  | t50_Farooq ~ ti(day_temp, night_temp, bs = "tp", k = 4) | 22.066 | 3.725 | 61.971 | 20.23077 | 0.5328257 |  |
|  | t50_Farooq ~ s(day_temp, night_temp, bs = "tp", k = 4) + s(day_temp) +     s(night_temp) | *NA* | *NA* | *NA* | *NA* | *NA* |  |
